# Relationship between serum cortisol levels, stereotypies, and the presence of autism spectrum disorder in patients with severe intellectual disability

**DOI:** 10.1038/s41598-024-57459-3

**Published:** 2024-03-26

**Authors:** Takeru Ohtsubo, Yoshito Mizoguchi, Chie Aita, Yoshiomi Imamura, Momoko Kobayashi, Yutaka Kunitake, Hiroshi Tateishi, Takefumi Ueno, Akira Monji

**Affiliations:** 1 Department of Psychiatry, National Hospital Organization Hizen Psychiatric Medical Center, 160 Mitsu, Yoshinogari, Kanzaki, Saga 842-0192 Japan; 2 Department of Psychiatry, Faculty of Medicine, Saga University, 5-1-1 Nabeshima, Saga, 849-8501 Japan; 3 Kyushu University, 744 Motooka, Nishi-ku, Fukuoka, 819-0395 Japan

**Keywords:** Intellectual disability, Autism spectrum disorder, Cortisol, Stereoty, Biochemistry, Psychology, Neurology, Signs and symptoms

## Abstract

Stereotypies are one of the diagnostic criteria for autism spectrum disorder (ASD) and are common to both ASD and intellectual disability (ID). Previous studies have been inconclusive, with some showing a positive correlation between stereotypies and cortisol, while others have shown a negative correlation. We hypothesised and investigated the presence of ASD as one of the variables involved in this discrepancy. We tested the following hypotheses on serum cortisol in a total of 84 hospitalised patients with severe ID and ASD with severe ID. Hypothesis (1) Higher levels of stereotypies are associated with higher levels of serum cortisol. Hypothesis (2) The presence of ASD will moderate the association between stereotypies and high serum cortisol levels. The results of the analysis supported hypotheses (1) and (2). We also found that in the population with ID, serum cortisol levels were significantly lower in the ASD group compared to the non-ASD group. The present findings that the association between stereotypies and serum cortisol levels in people with severe ID is moderated by the presence of ASD suggest that the stress response system may function differently in people with ID and ASD than in the general population.

## Introduction

Stereotypies are defined as repetitive movements or vocalisations that have no apparent function^1^. Examples include body tremors, hand flapping, repetitive vocalisations, spinning and banging of objects, with many variations^2^. Stereotypies are one of the diagnostic criteria for autism spectrum disorder (ASD)^3–5^ and are common to both ASD and intellectual disability (ID). However, the severity of the stereotypy is higher in children with ASD than in children with ID^6,7^. Stereotypies have a serious impact on an individual's daily life^8^. In addition, stereotypies may be maintained by internal rather than external stimuli, making them difficult to assess^9^ and burdensome for families and caregivers as a management challenge and barrier to adaptive learning^10–12^. Despite this, the precise neurobiological mechanisms involved in the maintenance of stereotypies in people with ASD and other developmental disorders are not well understood^13^.

One important neurobiological mechanism underlying stereotypy is the production of cortisol. Cortisol, also known as the stress hormone, is produced when a person is under stress and reflects the activity of the hypothalamic–pituitary–adrenal (HPA) system^14^. Previous neurobiological studies have not conclusively linked stereotypy to cortisol^15–17^. Animal studies have shown a positive association between stereotypy and cortisol^18,19^. However, in humans, some have found stereotypies to be associated with high cortisol levels^20,21^, while others have found them to be associated with low levels^22^. A number of variables may be involved in this discrepancy, including diurnal variation, the environment, the media from which cortisol is collected (e.g. blood, saliva, etc.), participant heterogeneity, and issues with the assessment method^15–17^. To eliminate the influence of variables, some attempts have been made to measure the area under the cortisol curve (AUC) and account for the effects of diurnal variation^21^, or to assess uniform participants by direct observation alone^17^. However, despite these measures, the results are still inconsistent, suggesting the influence of other variables not accounted for in previous studies.

Other studies have suggested that the presence of ASD itself is also a variable. For example, physical activity is known to reduce stereotypies without reducing other behaviours^23^, suggesting that stereotypies and physical activity may share some mechanisms. However, physical activity can reduce cortisol^24,25^ and stereotypies can increase cortisol as described above^20,21^. Although seemingly contradictory, the presence of ASD can be considered as an intervening variable. This is because, unlike studies of physical activity, most previous studies of stereotypy have been conducted with people with ASD; when comparing ASD and stereotypic development, it is difficult to treat ASD as a variable, but if the comparison is made within a group in which stereotypies are observed (e.g. an ID-only group versus an ASD with ID group), comparisons can be made.

As a mechanism by which stereotypies are maintained, the possibility that stereotypies in ASD may reduce stress levels also needs to be considered. Research has suggested that stereotypies in people with ASD may be a response to light, sound and sensory stimuli^26,27^. And it has been suggested that stereotypies in ASD may reduce stress and arousal levels caused by excessive sensory stimulation^28^. Animal studies also suggest that stereotypies may be a coping mechanism^29–31^. In interviews with people with high-functioning autism, responses indicate that stereotypies improve concentration, reduce external stimuli, reduce stress and lead to a sense of security^32^. It has also been shown that people with ASD with ID have unique coping mechanisms for acute stress^33^. It is possible that stereotypy may act as a coping mechanism, particularly for people with ASD.

Stereotypies are observed in people with both ID and ASD and might function as a coping mechanism to reduce or moderate cortisol levels. The purpose of this study was to measure serum cortisol in people with severe to profound ID and ASD in an inpatient hospital.

We then formulated and tested the following hypotheses:

(1) High levels of stereotypy are associated with high levels of serum cortisol levels;

(2) The presence of ASD moderates the association between stereotypies and high serum cortisol levels (i.e., in the absence of ASD features, high levels of stereotypies will be associated with high levels of serum cortisol).

## Materials and methods

### Study design and setting

This study was carried out according to the Strengthening the Reporting of Observational Studies in Epidemiology (STROBE) statement checklist. This study was approved by the Institutional Review Board of the Hizen Psychiatric Center (approval number: 2019-2) and was conducted according to Guidelines for Clinical Studies of the Japanese Ministry of Health, Labor and Welfare.

### Participants

Eligible participants were hospitalised patients of a special ward for severe intellectual disabilities at Hizen Psychiatric Center. The ward mainly admitted patients who were unable to live at home due to behavioural problems. Of the eligible participants, 84 patients (aged 16–60 years) took part in the study, all of whom underwent analysis of serum cortisol concentrations. All participants had severe or profound ID and almost all had an IQ of 35 or less. All participants were diagnosed with intellectual disability, and 56 of the 84 participants were also diagnosed with ASD according to the Diagnostic and Statistical Manual of Mental Disorders, fourth edition, text revision (DSM-IV-TR)^5^, and 36 participants were also diagnosed with epilepsy. Patients had no apparent psychiatric co-occurrence. All diagnoses were confirmed by a child psychiatrist (the first author) using the Childhood Autism Rating Scale-Tokyo Version (CARS-TV)^34^. Participants were unable to give informed consent due to their severe intellectual disabilities. Therefore, written informed consent was obtained from their legal guardians.

### Tests administered to all participants

Intellectual and developmental levels were assessed using the Tanaka-Binet Intelligence Scale V (fifth edition)^35^ and the Enjoji Scale of Infant Analytical Development^36^. The Tanaka-Binet Intelligence Scale was first published in Japan in 1947. This scale is suitable for people aged 2 years and over, including adults. The Tanaka-Binet Intelligence Scale V was published in 2005 and is a revised version of the scale for assessing development in children under one year of age and for measuring intelligence quotient (IQ) in adults. Deviation IQ (DIQ) scoring criteria, rather than conventional IQ measures, were calculated for participants over 14 years of age^35^.

The Enjoji Scale of Infant Analytical Development was developed in 1958. This scale is considered suitable for individuals from birth to 4 years and 7 months of age. Scores are obtained from observations and interviews with caregivers in six areas of functioning: locomotor movements, hand movements, basic habits, interpersonal relationships, language and natural language understanding, which are used to calculate the Developmental Quotient (DQ). Participants who could not be assessed using the Tanaka-Binet Intelligence Scale V were assessed using the Enjoji Scale of Infant Analytical Development^36^.

Stereotypies were assessed using the Japanese version of the Aberrant Behaviour Checklist (ABC-J)^37^. The ABC was originally developed to evaluate therapeutic effects in people with intellectual disabilities. The ABC-J is the Japanese version of the ABC and is considered to have the same factors and reliability as the original ABC, with analyses conducted on people with intellectual disabilities. The ABC and ABC-J consist of 58 items expressed as scores on five subscales: irritability, lethargy, stereotypic behaviour, hyperactivity and inappropriate speech. Of these subscales, stereotypic behaviour (stereotypy) was used in the present study.

### Cortisol assay

We obtained a blood sample from each participant at approximately the same time of day (13:00–14:00) over a 2-week period, as previously reported^38^. Blood cortisol concentration is reported to fluctuate depending on physical contact and serum cortisol levels show diurnal variation (early morning highs, nocturnal lows). Thus, we collected blood samples 1 h after participants consumed their midday meal, and these processes were performed before treatment with occupational therapy and play therapy. Before and after blood collection, no epileptic seizures observed. Blood samples (4 ml) were centrifuged at 2600×*g* for 15 min. Serum samples were stored in a freezer at − 80° until shipment, on dry ice, to the to the Saga University Faculty of Medicine for analysis. Assays were performed using an enzyme immunoassay kit (Cortisol Parameter Assay Kit, R&D Systems, Inc. USA). The inter-assay coefficient of variation was 13.6% and the intra-assay coefficient of variation was 7.0%, as reported by the manufacturer.

### Data analysis

All statistical analyses were performed using the Statistical Package for the Social Sciences (SPSS Statistics 23, IBM Japan, Tokyo). Initially, as a basic analysis, differences in gender, age, serum cortisol levels and stereotypies scores with and without ASD were examined. The χ-square test was used for gender differences.The normality of the distribution of serum cortisol levels was confirmed by drawing a histogram, so a t-test was used. The normality of age and stereotypies scores was not confirmed, so a Mann–Whitney U-test was performed in an independent sample. Next, a hierarchical multiple regression analysis was conducted with serum cortisol levels as the dependent variable for hypothesis 1 testing. The analysis procedure consisted of step 1 for the covariates gender and age, step 2 for the presence of ASD and stereotypies, and finally the interaction term of presence of ASD x stereotypy was entered into the regression equation. We followed Aiken and West^39^ and used values centred on the mean of the continuous variables, the ever-present behaviour and the dummy variable for the presence of ASD (individuals with ASD: 1, non-ASD individuals: 0). Hypothesis 2 was tested by simple slope analysis of the influence of an ASD diagnosis and engagement in stereotypy on serum cortisol levels.

## Results

### Basic analysis

We examined gender, age, serum cortisol and stereotypy scores according to the presence or absence of ASD (Table 1). The χ-square test and the Mann–Whitney U-test with independent samples were performed. We examined gender, age, serum cortisol and stereotypy scores according to the presence or absence of ASD (Table 1). The results of the χ^2^ test showed a significant gender bias, with more males than females in the ASD group (χ^2^ = 6.373 , df = 1, p = 0.012). Results of Mann–Whitney U-tests with independent samples showed that age (p = 0.002) and serum cortisol (p = 0.002) were significantly higher in the non-ASD group compared to the ASD group, and the ASD group had significantly higher values of stereotypy than the non-ASD group (p = 0.009). Serum cortisol was significantly higher in the non-ASD group than in the ASD group (t = 21.44, df = 35.17, p = 0.004), as a t-test was also performed to confirm normality.

**Table 1:** Comparison of clinical variables between ASD and non-ASD groups.

Clinical variable	Group	*n*	Means ± SD	Ranges	Mean rank	p-Value	Significant comparison
Age (years)	All	84	39.9 ± 11.69	16–60		*p* = .002	Non ASD > ASD
	ASD	56	37.2 ± 11.85	17–60	36.57		
	Non-ASD	28	45.4 ± 9.33	16–56	54.36		
Sex (male/female)	All	84	55/29			*χ*^2^(1) = 6.37,* p* = .012	ASD (male > female)
	ASD	56	41/15	–	–		
	Non-ASD	28	11/14	–	–		
Stereotypy	All	84	6.4 ± 5.45	0–21		*p* = .009	ASD > non-ASD
	ASD	56	7.3 ± 5.33	0–21	47.37		
	Non-ASD	28	4.5 ± 5.31	0–20	32.77		
Serum cortisol (ng/ml)	All	84	83.99 ± 26.917	1.206–205.880		*p* = .002	Non-ASD > ASD
	ASD	56	76.85 ± 18.754	1.206–131.040	36.57		
	Non-ASD	28	98.29 ± 34.541	46.902–205.880	54.36		

Reported values are means ± standard deviation (SD), ranges.

*ASD* autism spectrum disorder.

We found a positive correlation between the presence of an ASD diagnosis and the ABC stereotypic behavior domain scores (r = 0.241, p = 0.027) and a negative correlation between ASD and cortisol (r = -0.378, p < 0.001). There was no significant correlation between cortisol levels and stereoties (r = 0.122, p = 0.269).

### Hypothesis testing

We performed a hierarchical multiple regression analysis with serum cortisol levels as the dependent variable to test our hypotheses (Table 2).

**Table 2:** The results of hierarchical multiple regression analysis with serum cortisol concentration as the dependent variable (N = 84).

Independent variable	Step 1			Step 2			Step 3		
	*B*	*SE*	*β*	*B*	*SE*	*β*	*B*	*SE*	*β*
Sex^a^	−8.352	6.436	−0.148	−6.187	6.023	−0.110	−7.510	5.945	−0.133
Age	0.258	0.259	0.113	0.105	0.246	0.045	0.015	0.246	0.006
ASD/non-ASD^b^				−22.669	6.270	−0.399**	−25.223	6.280	−0.444***
Stereotypy				1.276	0.531	0.258*	2.776	0.908	0.562**
ASD × Stereotypy							−2.205	1.093	−0.357*
*R*^2^			0.036			0.203			0.243
Adjust *R*^2^			0.012			0.163**			0.194*
Δ*R*^2^						0.167**			0.040*

^a^Male = 1, female = 0.

^b^ASD = 1, non-ASD = 0.

*p < 0.05, **p < 0.01, ***p < 0.001.

The results showed that in step 2, the main effects of ASD presence/absence and stereotypy (in order; β = −0.399, B = −22.669, t = −3.616, p = 0.001; β = 0.258, B = 1.276, t = 2.402, p = 0.019) and finally the main effects of ASD presence/absence and stereotypy (in order; β = −0.444, B = −25.223, t = − 4.016, p < 0.001; β = −0.562, B = 2.776, t = 3.057, p = 0.003) and the interaction between ASD presence x stereotypy was significant (β = −0.357, B = −2.205, t = −2.017, p = 0.047). The R2 change for each step was significantly increased (step 2: p = 0.001; step 3: p = 0.047). The main effect of ASD presence showed that serum cortisol was lower the more ASD features were present. The main effect of stereotypy supported hypothesis 1 that the higher the level of stereotypy, the higher the serum cortisol level.

**Figure 1:**
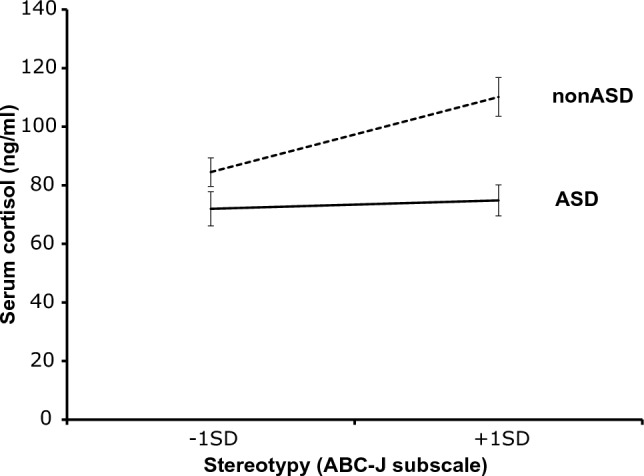
Interaction of stereotypy. Because the interaction term between ASD and stereotypy was significantly related to serum cortisol, we decided to further investigate the nature of the interaction between ASD and stereotypy according to a study by Cohen and Cohen^40^. The figure shows the substitution of ±1 SD of the value when ASD was set to 1 and non-ASD was assigned a value of 0, and ±1 SD of the value of stereotypy. The effect of stereotypy was significant for ASD (solid line). These results suggest that lower ASD scores are associated with a greater effect of stereotypy on serum cortisol levels. Thus, the association between stereotypy and serum cortisol levels differed between patients with and without ASD in patients with severe intellectual disability.

We then created the graph in Fig. 1 according to Cohen and Cohen^40^ to examine the nature of the ASD presence x stereotypy interaction that was significant in the final step. The obtained regression equation was created by assigning ASD = 1 and non-ASD = 0 to the presence/absence of ASD and ± 1 SD on average to the stereotypy. The figure shows the ± 1 SD of the values for ASD as 1 and non-ASD as 0, substituted for the ± 1 SD of the value of the ever-present behaviour. The effect of the stereotypy was significant in the case of non-ASD (dashed line) and not significant in the case of ASD (solid line).

**Figure 2:**
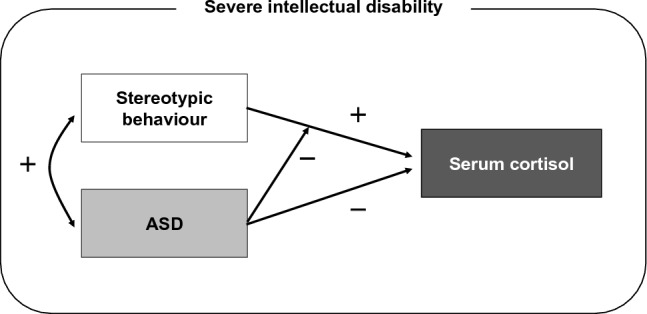
Relationships between stereotypies, serum cortisol and ASD. (1) There was a positive correlation between ASD characteristics and stereotypy scores; (2) higher ASD characteristic scores were associated with lower serum cortisol levels; (3) higher stereotypy scores were associated with higher serum cortisol levels; (4) the effect of stereotypy on cortisol levels varied depending on the presence or absence of ASD.

To test hypothesis 2, we examined whether the effect of the stereotypy on serum cortisol differed between the ASD and non-ASD groups. The results showed that the effect of the stereotypy on serum cortisol was significant in the non-ASD group (β = 0.532, B = 3.463, t = 2.798, p = 0.010) but non-significant in the ASD group (β = 0.122, B = 0.430, t = 0.861, p = 0.393). The non-ASD group showed that high levels of stereotypy promoted serum cortisol, while the ASD group showed no effect. The effect of stereotypy on serum cortisol levels was suggested to be modulated by the presence or absence of ASD, supporting hypothesis 2 (Fig. 2).

## Discussion

The present study examined the association between stereotypies and cortisol levels in people with severe ID; when ASD and non-ASD groups were examined separately, a correlation between high stereotypies scores and high serum cortisol levels was demonstrated. This supports hypothesis 1 and was similar to studies of stereotypies in animals^18,19^.Anumber of previous studies have examined the association between stereotypies and cortisol levels in ASD^15,16^. However, only a limited number have examined the association between stereotypies and cortisol levels in people with severe ID, with or without ASD. Symons et al. examined cortisol levels in a group of people with ID with and without chronic self-injurious behaviour (SIB) and found higher cortisol levels in the group with SIB^41^. Stereotypies and SIB are likely to coexist^42,43^ and there is a strong association between SIB and a range of stereotypies in people with ID^43^, which has parallels with the present study. The results of this study suggest that cortisol is important in inferring stereotypic tendencies in ID patients. However, larger studies are needed before cortisol can be validated as a biomarker for people with ID.

There have been several studies on how the presence of ASD affects cortisol levels, but nothing conclusive is known yet^15,16^. In addition, there are few studies comparing people with severe ID. The present study therefore investigated cortisol levels in people with severe ID in relation to the presence of ASD. The study is unique in that the developmental level of all participants (all diagnosed with severe intellectual disability) and their lifestyle rhythms, including sleep and diet, were consistent. The results showed that serum cortisol levels were significantly lower in the ASD group compared to the non-ASD group in people with severe ID; Tordjman et al. found that low-functioning autistic children had higher cortisol levels throughout the day than typically developing children^44^. Although this is not a direct link between our study and the comparison, because of the different comparisons, it is at least not contradictory. (For example, it could be hypothesised that cortisol levels in ASD with ID would be lower than in the ID-only group, but higher than in the typically developing group). In the future, if cortisol levels in ID, ASD with ID, high-functioning ASD and typically developing could be compared under the same conditions, it would be clearer how the presence of ID and ASD are related.

Another possible reason for the lower serum cortisol levels could be the effect of dysregulation of the HPA axis. The aforementioned study by Tordjman et al. found a significant flattening of cortisol levels, suggesting a slowing of circadian rhythms^44^. Other studies have also suggested dysregulation of the HPA axis in people with ASD with ID^15,16^; people with ASD often show effects even in normally non-stressful environments^45^ and have reported elevated stress levels compared to the general population^46–48^. Chronic stress can maintain high levels of circulating cortisol, cause brain damage such as hippocampal shrinkage, and impair the normal functioning of the HPA axis^49^

We investigated whether the effect of stereotypies on serum cortisol differed between the ASD and non-ASD groups. Results showed that higher stereotypies scores were associated with higher serum cortisol levels in the non-ASD group, but this effect was not observed in the ASD group. This supports hypothesis 2. To our knowledge, this is the first study to show that the effect of stereotypies on serum cortisol levels is moderated by the presence of ASD.From these findings, we considered two possibilities. The first is that the dysregulation of the HPA axis^15,16^ in ASD is also relevant here. The second is the possibility that stereotypies functioned as a coping behaviour, particularly in people with ASD, and were associated with lower cortisol levels than in people with ID only.

For the first, it is possible that higher chronic stress levels in people with ASD are associated with both more stereotyping^26,27^ and dysregulation of the HPA axis. If this is the case, it is possible that the effects of long-term dysregulation may be greater in those with more stereotypy in ASD, leading to suppressed cortisol release; Bitsika et al. found a positive correlation between stereotypy and basal cortisol levels in children with ASD, but not in adolescents^50^, who reported that this was not the case. This suggests that cortisol levels may change over time in individuals with high levels of stereotypy. Future additional longitudinal studies of stereotypies and cortisol levels in people with ASD that directly examine age-related changes would further support this hypothesis.

The second is the so-called self-regulation hypothesis. The theoretical support for the finding that ASD children with high stereotypies have low cortisol levels^22^ is the idea that this is a coping strategy of a group without appropriate coping strategies^51^. Tordjman et al. also focused on beta-endorphin (BE), a known brain chemical, and reported that it is higher in ASD with ID after stress^33^; BE is also released in stereotypies^52^, is released faster than cortisol^33^, and is known to be antagonistic to cortisol^53^. In other words, it may be possible to explain why people with ASD with ID are more likely to release BE and maintain (reinforce) stereotypies^54,55^, which in turn may alleviate cortisol elevations.

It is not possible at this stage to determine which of the two possibilities we have considered is predominant, and the possibility that both theories are valid cannot be ruled out. The present findings are consistent with many previous studies showing that the stress response system functions differently in people with ID and people with ASD with ID; further research is needed into the mechanisms involved in the maintenance of stereotypies in people with ID and people with ASD with ID.

### Limitations

The present study had several potential limitations. First, we did not diagnose ASD using the Autism Diagnostic Observation Schedule, Second Edition (ADOS-2) or the Autism Diagnostic Interview—Revised (ADI-R). This may have biased the diagnosis. The severity of the intellectual disability and, moreover, the difficulty in recalling the parents' old memories, precluded the use of these tools. The second is about blood sampling. In our ward, blood samples and other tests are regularly taken for the health care of the patients. The blood sampling in this study was done at the same time as the regular examinations. This minimised the additional burden on the patients participating in the study, but also made it difficult to make simple comparisons with many of the previous similar studies, such as those using saliva and early morning blood collection. Thirdly, it is about the participants. All participants in this study were institutionalised. This means that being institutionalised may have provided a constant living environment, while not being able to live with their families may have affected their stress and cortisol levels. In addition, the mean age of the ASD group was lower than that of the non-ASD group, and there were significantly more males than females in the ASD group.Epilepsy and other physical illnesses, as well as their treatments and antipsychotics, and self-harm as an Stereotypy also affect cortisol. However, relevant data were not sufficiently collected to be analysed. Furthermore, the analysis was inadequate in that it focused on the presence or absence of ASD rather than on the severity of ASD, and continuous variables could not be analysed.

## Conclusions

The present study investigated the relationship between serum cortisol levels and the presence of stereotypies and ASD in people with severe ID. The results showed that: higher levels of stereotypies were associated with higher serum cortisol levels. : the presence of ASD moderated the association between stereotypies and high serum cortisol levels. : also in the population with ID, serum cortisol levels were significantly lower in the ASD group compared to the non-ASD group. This suggests that the stress response system may function differently in people with ID and ASD than in the general population.

## Data Availability

The data supporting the results reported in our paper can be available from the corresponding author upon reasonable request.
